# Neonatal *Granulicatella elegans* Bacteremia, London, UK

**DOI:** 10.3201/eid1907.130009

**Published:** 2013-07

**Authors:** Leah Quartermain, Hiteshkumar Tailor, Salome Njenga, Parijat Bhattacharjee, G. Gopal Rao

**Affiliations:** Northwick Park Hospital, London, UK

**Keywords:** Granulicatella elegans, Abiotrophia, elegans, neonatal infection, bacteremia, nutritionally variant streptococci, bacteria, streptococci

**To the Editor:** *Granulicatella elegans*, a bacterium found in normal human oral flora, is generally associated with infective endocarditis. We discuss the identification and possible source of neonatal *G. elegans* bacteremia.

A 29-year-old woman sought care at Northwick Park Hospital (London, UK) at 41 weeks’ gestation (first pregnancy) for spontaneous rupture of membranes and discharge of clear liquor. She had fever (37.6°C) and a heart rate of 98 beats/min; there was no evidence of fetal distress. The woman was released from the hospital.

Twelve hours later, she returned because of discharge of meconium-stained liquor. Her white cell count was 18 × 10^9^/L (reference 3–10 × 10^9^/L), and her C-reactive protein level was 277 mg/L (reference <5 mg/L). Emergency cesarean section was performed after a diagnosis of fetal distress. A large amount of foul-smelling meconium was observed. A live male infant (3.05 kg) was delivered; Apgar score was normal. Blood samples were cultured for suspected sepsis, and the neonate was empirically administered intravenous benzylpenicillin and amikacin (6 days). He made a full clinical recovery.

The mother remained generally well, although she had persistent tachycardia (120 beats/min) and fever (37.6°C). She was intravenously administered amoxicillin/clavulanic acid and amikacin; over the next 2 days, her white cell count became normal, but her C-reactive protein level remained >400 mg/L. By postdelivery day 10, her temperature and heart rate were normal. Antimicrobial drug treatment was stopped, and she was released without further treatment. We interviewed the mother 8 months later and established that she had no dental procedures/infection or endocarditis before, during, or after pregnancy.

Placental swab samples were cultured on Columbia horse blood agar (CBA) and chocolated CBA (both incubated aerobically with 5% CO_2_ at 37°C for 24 hours), cysteine lactose electrolyte deficient agar (incubated in air at 37°C for 24 hours), and fastidious anaerobic agar with and without neomycin (incubated anaerobically at 37°C for 48 hours); all agar was from Thermo Fisher, Basingstoke, UK. On all media, the placental swab sample yielded moderate growth of tiny colonies, which Gram staining indicated were gram-positive coccobacilli.

Culture of the neonate’s blood sample (BacTalert 3D; Becton Dickinson, Oxford, UK) grew small, gram-variable bacilli after 17 hours of aerobic incubation. A subculture incubated aerobically on CBA or chocolated CBA showed no bacterial growth; however, tiny colonies were seen on fastidious anaerobic agar with and without neomycin. Gram staining of the colonies showed gram-positive bacilli that were morphologically similar to those isolated from placenta. We suspected lactobacilli or streptococci, but testing (API Strep and Coryne strips; bioMérieux UK Ltd, Hampshire, UK) did not confirm this. Nutritionally variant streptococci were not suspected.

Both isolates were sent to the Health Protection Agency Laboratory of Healthcare Infections (London). Antimicrobial drug sensitivities were determined (Iso-Sensitest Agar; Thermo Fisher) according to a previously defined method (http://bsac.org.uk/wp-content/uploads/2012/02/Version-11-2012-Final-.pdf). The isolates were sensitive to penicillin, clarithromycin, trimethoprim, and vancomycin but resistant to tetracycline. Partial sequencing of the 16S rRNA genes of both isolates confirmed they were the same *G. elegans* strain. Isolates exhibited enhanced growth in the presence of pyridoxal (Figure) and satellitism with *Staphylococcus aureus*.

**Figure:**
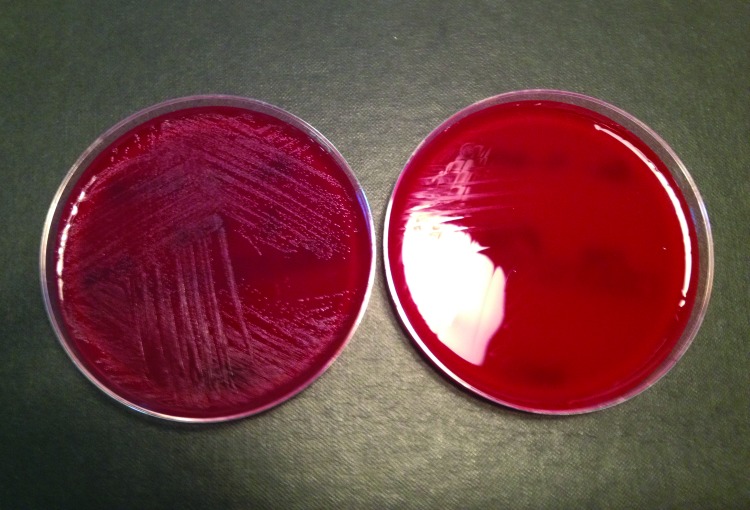
Blood agar plates with (left) and without (right) pyridoxal supplement from a study of neonatal *Granulicatella elegans* bacteremia, London, UK.

*G. elegans* (originally known as *Abiotrophia elegans*) was first described in 1998 (*1*) as a catalase-negative, oxidase-negative, nonmotile, facultative anaerobic gram-positive bacterium. However, the bacterium can exhibit variability and pleomorphism on Gram staining: forms range from bacilli in nutrient-depleted media to cocci arranged in short chains in nutrient-rich media (*1**,**2*). This variability poses challenges to the identification and taxonomic classification of the organism. The possibility of nutritionally variant streptococci (NVS) should be considered when gram-positive cocci or bacilli are seen on Gram staining, but the cocci/bacilli grow poorly on nonsupplemented media. Studies have shown that pyridoxine facilitates the growth of NVS (*3*).

Antibiotic susceptibility tests for NVS should be performed on media supplemented with pyridoxal (*3*). NVS are usually susceptible or moderately susceptible to penicillin. Strains tolerant to penicillin have also been reported, especially in the presence of supplements (e.g., pyridoxal and cysteine). High-dose penicillin and aminoglycoside are recommended for the treatment of serious NVS infections (*3*).

*G. elegans* bacteria can be part of normal oral flora (*4*); however, the bacteria are predominantly isolated from blood cultures for patients with infective endocarditis. Gonzales-Marin et al. (*5*) detected *G. elegans* in nasogastric isolates of neonates. The authors concluded that hematogenous translocation of maternal oral flora into the amniotic environment was the likely source because, the authors stated, *G. elegans* is not part of the normal vaginal flora. However, *G. elegans* has been isolated from the vaginal tract of healthy women (*6*).

Early-onset neonatal bacteremia caused by *G. adiacens* has also been reported (*7*). Molecular studies identified the same organism in the maternal cervical flora, suggesting ascending infection or acquisition of the bacteria by the neonate during delivery.

We cannot be certain whether the neonatal *G. elegans* bacteremia was caused by ascending or transplacental infection. We think the former is more likely in the light of the relatively prolonged rupture of membranes and the presence of chorioamnionitis.
